# On the Relationship Between EB-3 Profiles and Microtubules Growth in Cultured Cells

**DOI:** 10.3389/fmolb.2021.745089

**Published:** 2021-11-08

**Authors:** Arshat Urazbaev, Anara Serikbaeva, Anna Tvorogova, Azamat Dusenbayev, Sholpan Kauanova, Ivan Vorobjev

**Affiliations:** ^1^ National Laboratory Astana, Nazarbayev University, Nur-Sultan, Kazakhstan; ^2^ Laboratory of Biophotonics and Imaging, National Laboratory Astana, Nazarbayev University, Nur-Sultan, Kazakhstan; ^3^ Department of Physiology and Biophysics (M/C 901), University of Illinois at Chicago, Chicago, IL, United States; ^4^ A.N.Belozersky Institute of Physico-Chemical Biology, Lomonosov Moscow State University, Moscow, Russia; ^5^ Department of Biology, School of Sciences and Humanities, Nazarbayev University, Nur-Sultan, Kazakhstan

**Keywords:** microtubules, dynamic instability, EB proteins, fluorescent microscopy, cell culture, live cell imaging

## Abstract

Microtubules are dynamic structures undergoing rapid growth and shrinkage in living cells and *in vitro*. The growth of microtubules *in vitro* was analyzed with subpixel precision (Maurer et al., Current Biology, 2014, 24 (4), 372–384); however, to what extent these results could be applied for microtubules growing *in vivo* remains largely unknown. Particularly, the question is whether microtubule growth velocity in cells could be sufficiently approximated by a Gaussian distribution or its variability requires a more sophisticated description? Addressing this question, we used time-lapse microscopy and mathematical modeling, and we analyzed EB-3 comets forming on microtubules of cultured cells with subpixel precision. Parameters of comets (shape, form, and velocity) were used as topological characteristics of 3D voxel objects. Using regression analysis, we determined the real positions of the microtubule tips in time-lapse sequences. By exponential decay fitting of the restored comet intensity profile, we found that *in vivo* EB-3 rapidly exchanges on growing microtubule ends with a decoration time ∼ 2 s. We next developed the model showing that the best correlation between comet length and microtubule end growth velocity is at time intervals close to the decoration time. In the cells, EB comet length positively correlates with microtubule growth velocity in preceding time intervals, while demonstrating no correlation in subsequent time intervals. Correlation between comet length and instantaneous growth velocity of microtubules remains under nocodazole treatment when mean values of both parameters decrease. Our data show that the growth of microtubules in living cells is well-approximated by a constant velocity with large stochastic fluctuations.

## Introduction

Microtubules (MTs) are polymers of α/β-tubulin dimers that exhibit dynamic instability behavior (Mitchison and Kirschner, 1984), with their plus ends frequently switching between growth and shrinkage phases. MT dynamics *in vivo* are generally reported by six parameters: the rates of growth and shortening, duration of the attenuated state (pauses), and the frequencies of switching between these three phases. Tracking of growing MTs in cells using fluorescently labeled tubulin is a challenging task because of the relatively high density of these structures in the cytoplasm (Odde, 1997; Vorobjev et al., 1999). The alternative strategy was developed by using fluorescently labeled plus end–tracking proteins (+TIPs; Schuyler and Pellman, 2001)*.* Fluorescently tagged EB proteins bound to the ends of growing MTs and appear as comet-like structures that could be traced in time-lapse experiments with high precision (Bieling et al., 2007; Seetapun et al., 2012; Maurer et al., 2014).

EB proteins turn over on growing MT ends and forms characteristic cap there during the MT growth period (Dragestein et al., 2008; Duellberg et al., 2016; Guesdon et al., 2016). These proteins bind exclusively to the growing MT plus ends and are best suitable to examine MT dynamics *in vivo* (Galjart, 2010; Matov et al., 2010). EB intensity profile on the MT tips represents a cap, made of GTP-bound tubulin subunits (Bowne-Anderson et al., 2013; Brouhard and Rice, 2018). This profile is approximated with an exponential decay to extract the characteristic comet length–L (Bieling et al., 2008; Seetapun et al., 2012; Maurer et al., 2014). *In vitro* EB cap size is proportional to the rate of MT elongation (Rickman et al., 2017) and thus could be used for indirect measurements of the MT growth rate. However, analysis of MT dynamics *in vivo* does not consider the precise structure of the plus end comet (Matov et al., 2010), and the results of measurements are mean velocity. When measurements of the plus end displacement are made at short time intervals (to determine instantaneous growth rate), the accuracy of such measurements is doubtful, unless the position of the MT tip is determined with subpixel precision. Taking this into account, we recently conducted a detailed analysis of the comet length measurement and show that EB comets could be described by a piecewise exponential/Gaussian function approximation (Mustyatsa et al., 2019). The exponential/Gaussian function approximation or Gaussponent method allows achieving the sub-pixel precision obtained at *in vitro* measurements. Thus, the use of the comet length might be beneficial for MT dynamics analysis, but to use it, one needs to minimize the limitations of such analysis. In the current study, we have undertaken the analysis of the correlation between the size of EB-3 comets and MT growth velocity in the cancer cells. We developed the model allowing precise determination of the comet length and head position considering the shift of the start (zero points) of exponential decay from the brightest point on the profile. Our data show that EB-3 rapidly accumulates on the growing MT end with a short decoration time. The growth of a microtubule in a cell can be described as a process going on at a constant rate with random fluctuations.

## Results

**FIGURE 1:**
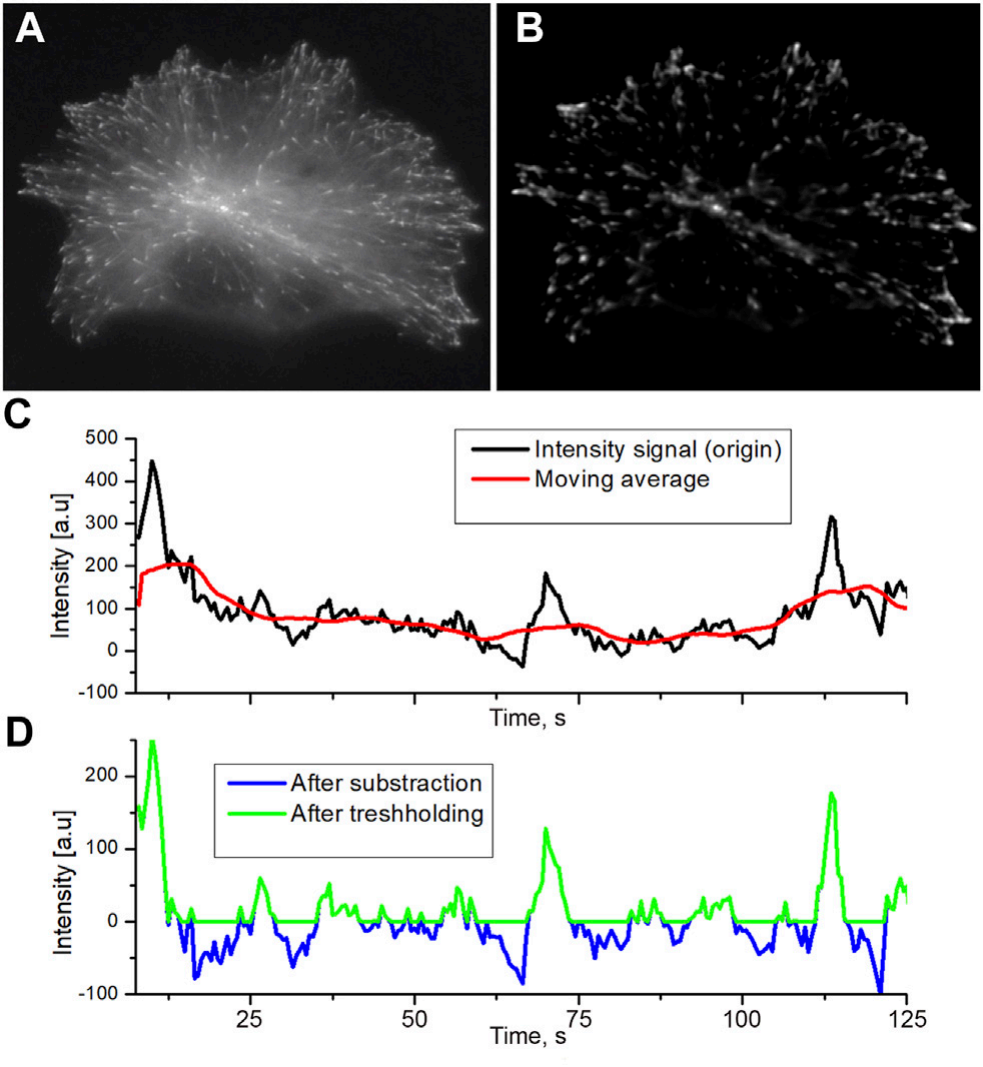
Raw initial image of EB comets in the transfected HT-1080 cell **(A)** and normalized image by the running averaging and thresholding applied **(B)**. The background in the normalized image is set to 0 intensity. Original profiles and moving average filtering **(C)** black line: original intensity, red line: the result of moving average. The thresholding of intensity after subtraction of the moving average **(D)** is marked by a blue line. Green line: intensity after thresholding. All negative values were assigned to zero.

We performed a live cell imaging experiment to collect images of the growing microtubule ends (the MT “comets”) labeled by EB-3-RFP fluorescent probes with a time resolution of 500 ms. The collected sequences of the comet’s images were used to describe the fine structure of EB-3 comets and to determine the dependence of the length of the comet tail on the velocity of displacement of the growing MT. (Figure 1). To further measure the comet size, velocity, and shape, we developed an automated analytical routine.

Processing software consists of two parts:

1) A comet tracker for the detection of the growing MT tip based on a topology algorithm. A tracker code was developed in MATLAB, and the function of the flood fill algorithm was developed in c++. This part of the program tracks the comets, builds trajectories for comets, and filters comets from other objects in image sequences.
2) A comet tail analyzer to extract the tail length from the data of the tracked comets. This part was developed on MATLAB. The comet tail analyzer allows extraction of the 2D profile of comet intensity (from head to tail) and making regression with the convoluted function of brightness. It allows getting the tail length of a comet.

### Image Preprocessing for Automated Analysis of the “Comets”

The original raw image of MT comets contains noises, uneven illumination, and unwanted autofluorescent particles that need to be removed (Figure 1A). The preprocessing protocol we developed exploits spatial and time properties of growing comet objects to remove unwanted background pollution.

At the initial step, images were filtered by Fourier transformation (both high pass and low pass) to reduce noises. High-pass filtering reduces illumination unevenness. The low-pass filtering suppresses the high-frequency noises generated by the detector matrix (Figure 1B). Denoised images obtained could be described as 2D matrix, where columns are y coordinate, rows are x coordinate, and the intensity of a pixel will be the value of matrix cell I (x, y). These images were further used to render a 3D data matrix I (x, y, t), where I (x, y) is the intensity matrix of microscope images for the time point t as a 4^th^ semi-dimension. Thus, we get the sequence I (t) for each point with spatial coordinates x, y. The running average with 30 time points was used to produce an array I_s_ (t) of local n-point mean values, where each means is calculated over a sliding window of length *n* across neighboring elements of I(t).

When *n* is odd, the window is centered about the element in the current position. When *n* is even, the window is centered on the current and previous elements. The window is automatically truncated at the endpoints when there are not enough elements to fill it. When the window is truncated, the average is taken over only for the elements that fill the window. I_s_ (t) has the same size as I (t).

The final operation was the subtraction of I_s_ (t) from an original sequence I (t). This subtraction keeps only objects changing in the time domain such as comets, while static objects are wiped off. After subtraction, the average background intensity becomes about zero or negative, and background pixels have negative values (Figure 1C, blue profile). To remove residual noise from the image without affecting comet intensity profiles, the threshold bringing all negative values to zero was applied (Figure 1D).

When the intensity of the comet against the background is high, the level of the threshold will be less than 10% of the average level of the comets. Comets with moderate and low intensity, where thresholding cut a significant part of the profile, were not analyzed further.

### Topological Comet Tracker Based on Gradient and Flood Fill Method

**FIGURE 2:**
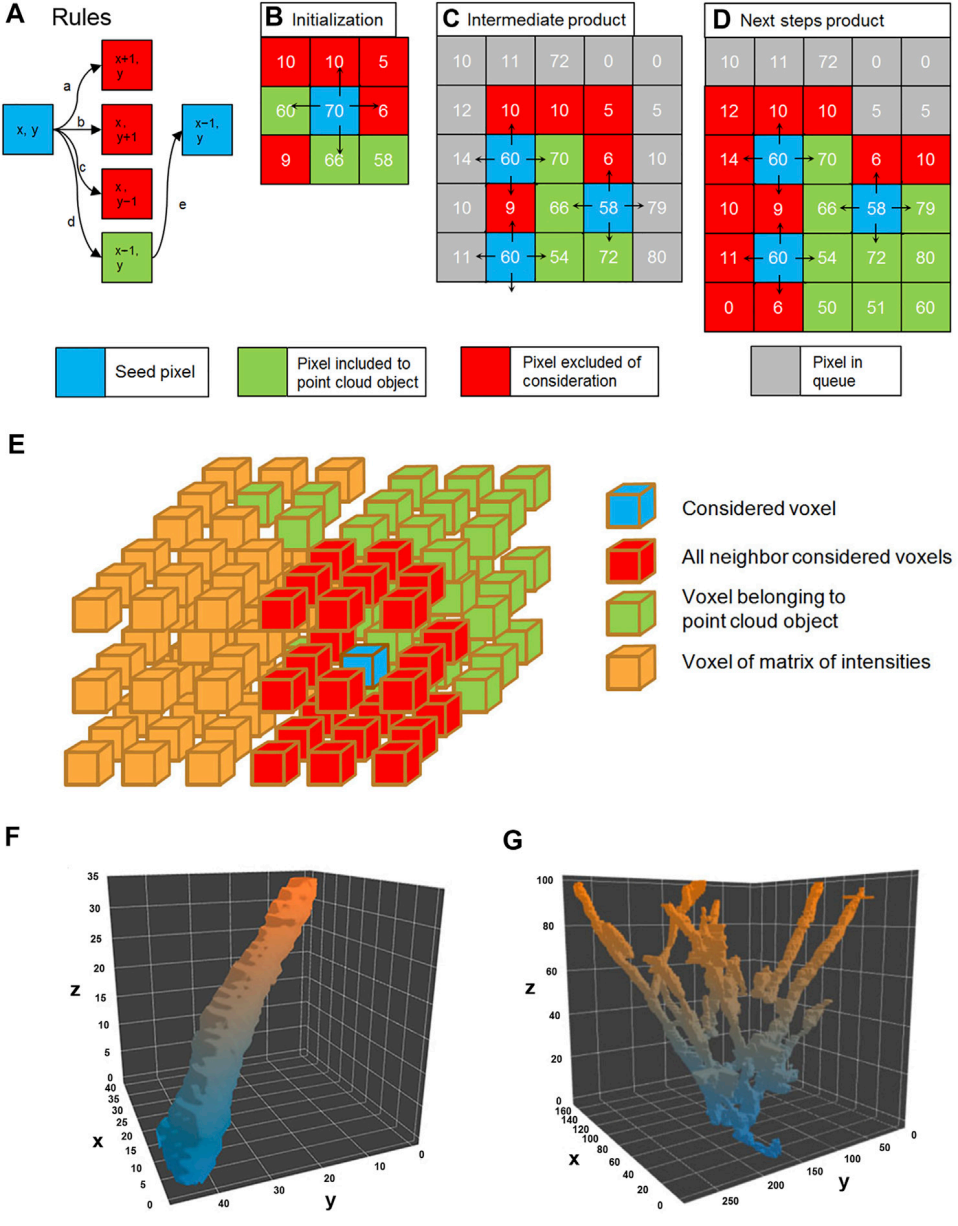
Flood fill algorithm procedure rules **(A)**. The only pixel which satisfies the rule (marked green) is chosen as seed to the next step marked green. The initialization of flood fill by seed pixel **(B)**; in the consecutive steps **(C)** and **(D)** flood fill a search for other pixels satisfying the rules. The 3D representation of the flood fill algorithm **(E)**. The result is 3D visualization of the plus end comet in the spatial–temporal domain **(F)**. A pillar represents the trajectory of the segmented growing single microtubule, and the Z domain is time. The multitude of growing microtubules forms an object point cloud where comets form a tree shape, where comets’ tracks may overlap **(G)**.

To find a correlation between tail length and velocity, our growing MT end tracker treats each comet as a 4D object, where the third spatial dimension is time and intensity is in a hidden 4th dimension, as described in the previous section. We next applied a flood fill algorithm to detect all bright objects in the stacks based on the gradient analysis. The comet pixel profile consists of pixels with brightness above the background and using the differences of intensities between adjacent pixels this algorithm determines a margin of comet head. The initial “seed” pixels were taken as having significant brightness, and a pixel cloud was formed around them (Figure 2A–D) for each comet. Object’s edge was determined as forming a large gradient with the local pixel neighborhood (Supplementary Material S1).

Clouds of positive voxels were determined, and 3D profiles of the comets were generated (Figure 2E). Each comet is presented as a 4D object: spatial g (x, y, z), where z represents time (t) and I (intensity) is a “hidden” dimension. Since the comet is moving in time and space domains in the 3D representation (x, y, t), it appears as an elongated pillar (Figure 2F). The length of the pillar reflects the life span of the comet.

However, not all pillars represent individual comets—some comet’s tracks collide with each other because Airy disks of the closely located MT tips overlap, and residual non-comet objects might be present (Figure 2G).

**FIGURE 3:**
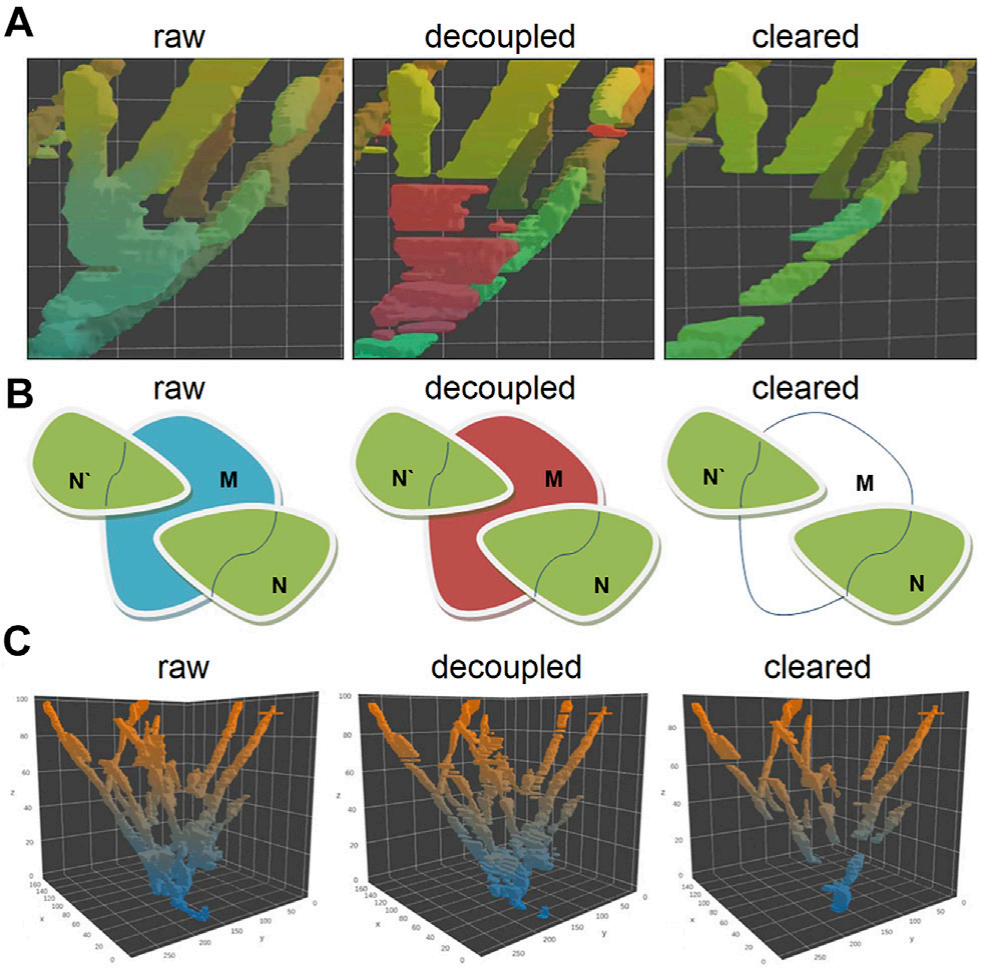
Visualization of the plus end comet collision in a spatial–temporal **(A)** and 2D projection of the collision point **(B)**, before decoupling and after removing all non-comet objects. The decoupling algorithm compares the topological sets of XY coordinates with each other in T_n_–T_n+1_ for a given tree object of objects N and N′ shift toward each other along the *Z*-axis form merged intersection object M at a certain point of time. The merged tracks of comets M belong to the T_n+1_ plane, track N, and track N′ belong to T_n_. This object can be represented as a set M in T_n+1_ and as the sets N and N′ in T_n_ for the same X-Y coordinates. So, if M ∩ N′ and M ∩ N are non-empty sets and if the number of these non-empty sets are 2 or greater (for a greater number of comets), this means collision of comets occurs. For such cases, M set is removed from the voxel cloud and splits into several pieces. The 3D visualization of the decoupling process of the topological object point cloud **(C)**: after decoupling of the comet collision, event the solid “tree” shape object converts into a set of separate comet tracks.

Since the distance between growing MT ends might be well below the resolution of the light microscope (Airy disk radius), we need decoupling to make sure that automated analysis is always assigned to individual tracks without jumping from one track onto another. To remove apparently colliding comets from further analysis, we decomposed obtained point cloud into single comet tracks by the “decoupling” method based on the topological structure of the cloud and determining features of comet tracks (lifespan, size, and intensity) (Figure 3). We removed all planes where pillar fusion (comet apparent collision) happened from the further analysis and dissected each object tree into several pieces, where each piece reflects a single comet track or part of the track (Figure 3B–C).

The other non-comet objects were removed by analyzing the binary point cloud object mask in the 3D space. Since the comet moves in the (x, y, t) domain (where t means time), it forms the pillar structure with a certain “tilt” to the *z*-axis in a 3D coordinate cube. The objects with a small angle (nearly vertical) or occupying not more than three consecutive slices along the time axis were excluded.

Besides, we considered the presence of the noise and removed from further processing small objects with the integral size, below than 500 voxels, or the cross-section in the T_n_ smaller than 50 pixels (in one X–Y plane). Only pillar objects with relatively large cross sections and going through at least four consecutive X–Y planes were left for further analysis (Figure 3C, cleared). These binary masks are the tracks of individual comets allowing estimation of the velocity of MT growth. The overall number of individual comets used for further analysis was 31,623 in control HT1080 cells and 6,640 among the cells treated with nocodazole. A detailed analysis of the comet length and velocity is given further.

### Calculation of the Comet Length

**FIGURE 4:**
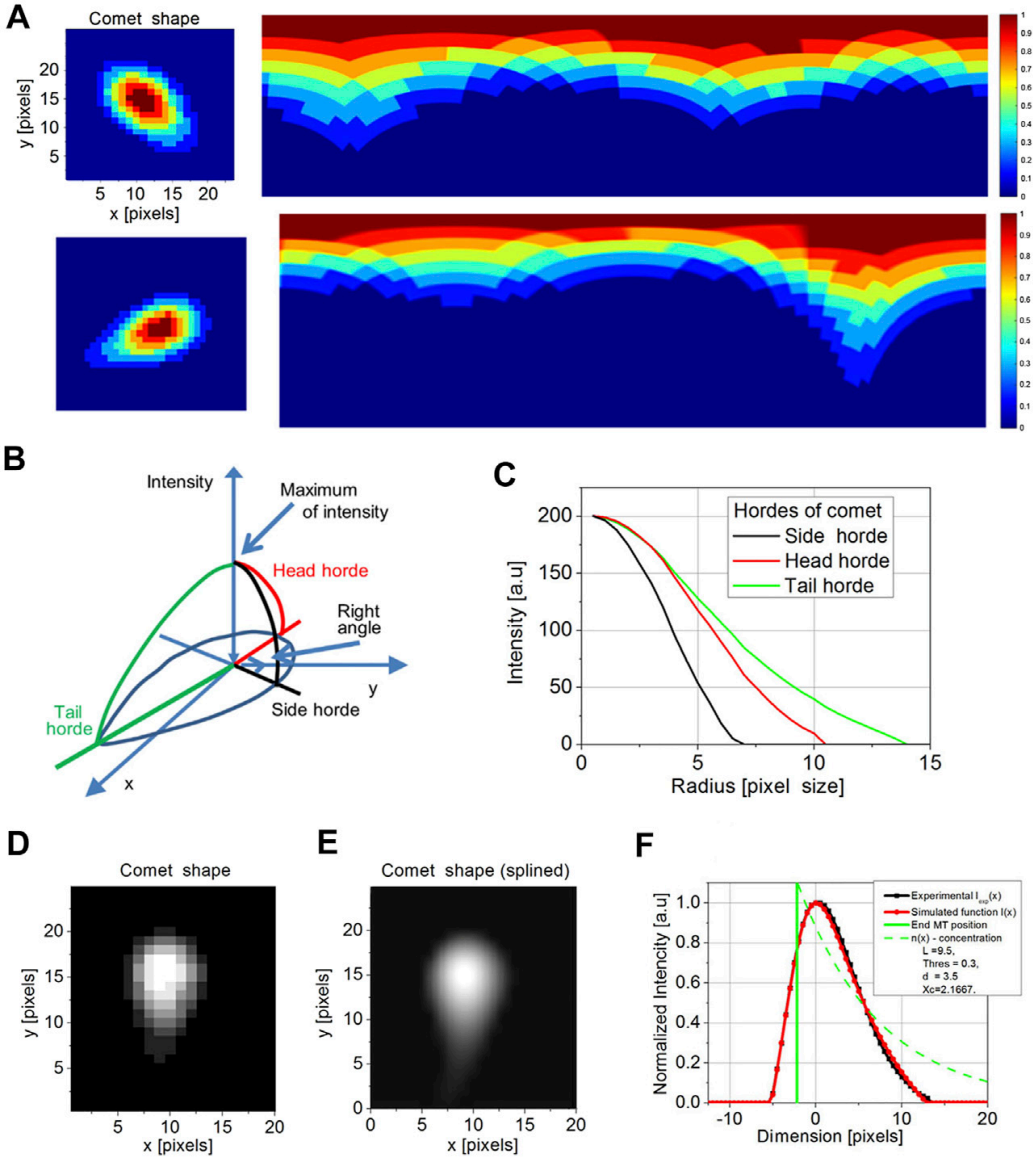
Cartesian to polar transformation of a comet in a single time point for the comet with a short and long-tail **(A)**. The initial coordinates for transformation are set by maximum intensity pixel. The rainbow scale bar represents the intensity of each pixel. The model of comet intensities in XY and I (intensity) representation **(B)** was used to determine side (black) head (red) and tail (green) hordes. The model hordes of side (black), head (red), and tail (green) **(C)** were then compared with comet profiles. The original comet image is pixelized **(D)**. To compare it with a model, we perform a spline of comet images **(E)**. After determining the tail (red) and the real position of the tip of the microtubule (maximum in green), the comparison of exponential regression obtained by the current model (green), experimental profile (black), and simulated profile (red) was performed **(F)**. There is a significant shift between the MT end and an apparent maximum of intensity (set to 0).

Comet in a digital image is a 3D object with the XY coordinate and I intensity dimension. We assume all “true” comets having pixel profiles close to a cabochon shape with a roundish “head” and elongated “tail.” The growing MT itself is a quasi-1D object. Therefore, there is no need to consider a 3D object. It is enough to consider a 2D object, which is a slice of the original object by a plane passing along the tube itself. It will also simplify calculations. The first step is to convert an image of this 2D object from Cartesian (x, y) to polar coordinates (r, ϕ), where the pole of new coordinates is located at the maximum of the comet intensity (Figure 4A). We assume that the center of intensity lies on the microtubule. Then we applied 64 angular profiles (“hordes,” with a step Δϕ = 5.625) to determine the comet long axis direction (Figures 4B,C). To smooth the transition between coordinate systems, we used 10x rescaling by cubic splining (Figure 4D). As we mentioned before, the high- and low-pass Fourier filters were used. Therefore, our profiles are already “smooth,” and using cubic spline routing does not cause a problem with additional mode spawning. Profiles of intensity were determined along with each horde. The comet “tail” horde is taken as the function with a maximal integral value. Comet “head” horde is determined as opposed to the tail horde, and the comet “side” hordes are determined as perpendicular to the direction of comet “tail”.

We detect the length of the tail (comet length) from the entire profile of each comet formed by detecting the intensity signal produced by EB-3–RFP probe proteins (Figure 4F). According to the existing model of EB accumulation, the distribution of the EB-3 protein on the tip of the growing microtubule should have exponential decay due to a decrease in the number of EB-3–binding sites along growing MT (Bieling et al., 2008; Duellberg et al., 2016). Thus, the comet’s tail intensity profile is as follows:

$$n\left(x,t\right)=N\left(t\right)\text{∗}e^{-\frac{x}{L}},$$

where n (x, t) is a function of light intensity, N (t) is time dependent on the function that is considered at a certain time point, N (t) remains constant (A), and L is the required comet tail length:

$$n\left(x\right)=A\text{∗}e^{-\frac{x}{L}}.$$

We model the comet intensity profile acquired by the sCMOS detector array as the sum of Airy disks generated by individual EB-3-RFP molecules. It is denoted as I_exp_ (x_k_).

The intensity of light I in the comet profile is a convolution of n(x) with PSF (point spread function) of the microscope (Maurer et al., 2014) that is well-approximated by Gaussian function (Zhang et al., 2007):

$$\mathrm{PSF}\left(x\right)=\;e^{-\frac{x^{2}}{d^{2}}},$$

where d of Gaussian is the width of PSF.

In our model, *d* is determined for each comet from the further regression analysis. The variable value of *d* represents slight defocusing of the comet images obtained by wide-field microscopy.

The real position of the comet tip (x_c_) is shifted from the maximum of intensity in the microscopic image (Maurer et al., 2014). The shift between maximum intensity position and concentration function n(x) denoted as x_c_ is described in simulation function:

$$\{\begin{array}{c} n\left(x\right)=A*e^{-\frac{x-x_{c}}{L}};\;x>x_{c}\\ n\left(x\right)=0;x<x_{c} \end{array}.$$

Total comet profile intensity will be the product of convolution:

I(x)=(n(x)∗PSF(x))(x).

And, since n(x) and PSF depends on the intensity function,

$$I\left(x\right)∼\;\underset{x_{k}}{\sum }n\left(x_{k}\right)\text{∗}e^{-\frac{{\left(x-x_{k}\right)}^{2}}{d^{2}}}.$$

Another important parameter in the model is T (threshold) used to cut off pixels with low intensity and assigning their values to zero (Figure 1D). Thus, we added a condition related to the thresholding operation:  if I(x)<0 then I(x)= 0; as mentioned earlier, we used time averaging and thresholding in preprocessing of images. In our way of preprocessing, the threshold is a slowly changing function and for different comets is not constant. Threshold affects the shape of the intensity curve, and we considered it. Thus, to increase fitness accuracy, we added a condition related to the thresholding operation:  if I(x)<0 then I(x)= 0.

Using $T,\;x_{c},\;d,\text{ and }L\;$ as variables, we perform fitting of the real comet profile with the simulated one. The simulated profile was constructed as follows:

$$\{\begin{array}{c} I\left(x\right)=\sum_{x_{k}}n\left(x_{k}\right)\text{∗}e^{-\frac{{\left(x-x_{k}\right)}^{2}}{d}}-T\;\\ \;and\;if\;I\left(x\right)<0\;than\;I\left(x\right)=\;0 \end{array}.$$

To find the best fit values of $T,\;x_{c},\;d,\text{ and }L\;$ for a comet, the regression with the experimental function I_exp_ (x_k_) was performed:

$$\left(T,\;x_{c},\;d,\;L\;\right)=\arg\underset{T,\;x_{c},\;d,\;L}{\min}\left(\sum_{k}{\left(I\left(x_{k}\right)-I_{\exp}\left(x_{k}\right)\right)}^{2}\right).$$ (4)

By the regression analysis, we obtained the following comet features: the threshold weight T, x_c_ that is the coordinate of the MT end in the local system of coordinates, *d* is the blur of PSF, and finally, L is the length of comet tail (distance where intensity drops down e times from the maximum). Reconstruction of the intensity profile of a comet is shown in Figure 4F. Two parameters are taken for further consideration: position of the MT end (x_c_) and comet length (L).

The intensity maxima of comets had been obtained from local polar coordinates (x_c_) was then returned to the global Cartesian coordinate system:

$$\{\begin{array}{c} x_{\mathrm{end}}=x_{\max}+x_{c}*cos\left(\varphi_{\mathrm{head}}\right)\\ y_{\mathrm{end}}=y_{\max}+x_{c}*sin\left(\varphi_{\mathrm{head}}\right) \end{array},$$

where ( $x_{\max},y_{\max})$ are coordinates of the maximum of the intensity of comet in a global system of coordinates and $\varphi_{\mathrm{head}}$ is the angle of the head horde.

### Measuring the Velocity of the MT Growth

**FIGURE 5:**
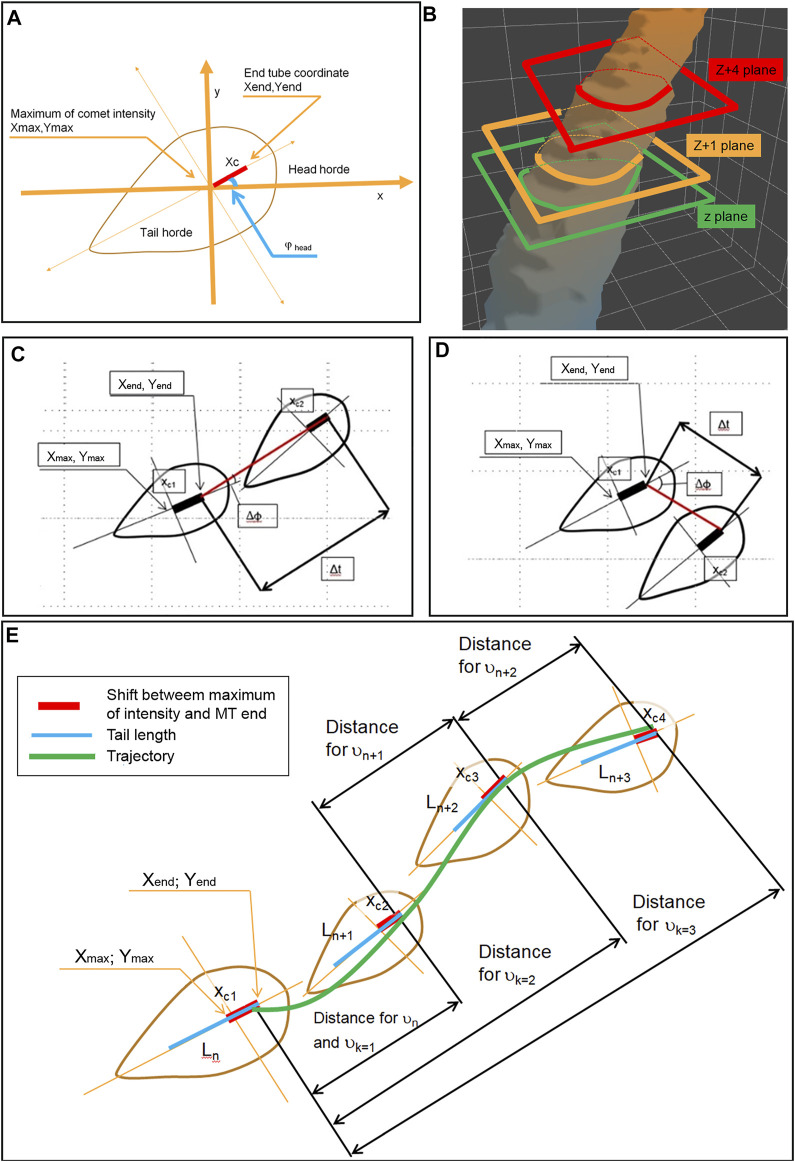
Determination of the MT growth trajectory and measurements of the velocity by determination of the head horde and scheme of MT end coordinate measurements **(A)** and by neighbor planes, z and z+1, relates to time t and time t+1 on the topological object **(B)**. The plane z+4 relates to t+4 in case when the direction of a comet (head-to-tail axis) is close to MT growing direction (red line) **(C)** and in case where the direction of a comet is different from MT growing direction **(D)**. **(E)** The velocity measurements performed on the comets with both directions coinciding (with L_n_, L_n+1_, L_n+2_, and L_n+3_ were measured sequentially). The distances for the measurement of *v*_n_, *v*_n+1_, and *v*_n+2_ are shown. Also, distances for calculation *v*_k=1_ *v*_k=2_ *v*_k=3_ are shown. The green line shows the splined trajectory of the MT growth over few frames.

For the determination of the velocity of MT growth, we next analyze comet head displacement between successive frames. The glowing comet appears trembling on the sequence of images while passing through the interior of the cell. It might represent ambiguity of our measurements due to “fishtailing” (lateral displacement) of the MT ends. In manual measurements, by connecting the consecutive comet head positions with a straight line when time discrete is short (<2–3 s) frequently gives relatively high angles between comet long axis and comet displacement vector (Figures 5A,B). This non-axial displacement might cause an overestimation of true MT growth events if the angle is too big (Figures 5C,D). The tracking algorithm keeps the only comet within a 35°angle between the displacement vector and comet axis (Figure 5E).

The “instantaneous” velocity ***ϑ*** is measured as a distance between MT ends of the tracked comet between the z plane and z+1 plane:

$$\vartheta =\frac{\sqrt{{\left(x_{1}-x_{2}\right)}^{2}+{\left(y_{1}-y_{2}\right)}^{2}}}{t}=\frac{\sqrt{\left(x_{\mathrm{max1}}-x_{\mathrm{xmax2}}+{\left({\text{x}}_{\text{c}1}-{\text{x}}_{c2}\text{*}\cos\left(\varphi_{\mathrm{head}}\right)\right)}^{2}+{\left(y_{\max1}-y_{\mathrm{xmax}2}+\left({\text{x}}_{\text{c}1}-{\text{x}}_{c2}\right)\text{*}\sin\left(\varphi_{\mathrm{head}}\right)\right)}^{2}\right)}}{t}\;\text{.}$$

**FIGURE 6:**
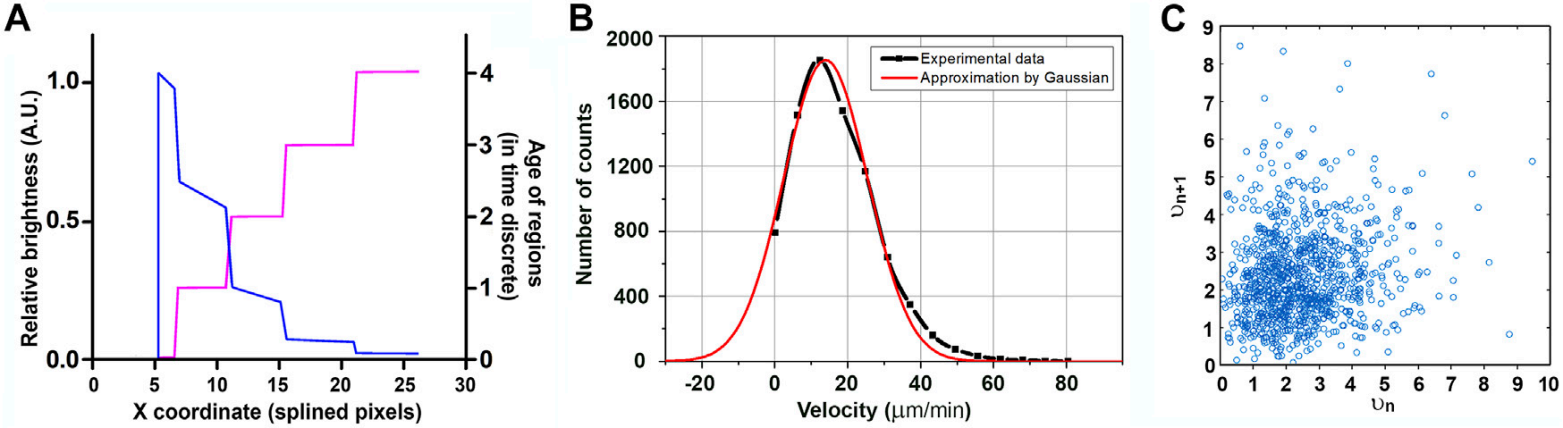
EB-3 intensity by piecewise approximation. The older the ROI, the lower is the signal in it. A signal is recorded intermittently, and piecewise approximation represents the expected intensity distribution within the comet if the affinity of the MT to EB-3 decreases exponentially with time (blue line) **(A)**. The “age” of regions depended on the x coordinate for the microtubule (purple line). **(B)**. MT velocity histogram for HT 1080 cells approximated by Gaussian distribution. *Y*-axis: comet counts. µ = 13.9 µm/min; σ = 16.3 µm/min; R^2^>0.98. **(C)** Lack of correlation between growth velocities (given in A.U.) in subsequent time periods (*v*_n_ versus *v*_n+1_).

For different time intervals, we obtained growth velocity distributions for both cell lines close to the Gaussian one (Figure 6C), which means the elongation of MTs in these cells could be approximated as growth with constant velocity (with random normal noise). A MT velocity histogram for HT 1080 cells is approximated by Gaussian distribution, with µ describing the Gaussian maximum coordinates and σ the function width at the decrease in e time. Growth velocity for HT-1080 is µ = 13.9 µm/min; σ = 16.3 µm/min (large σ reflects the presence of negative velocities (see Maurer et al., 2014 for more details). It is important to notice that instantaneous velocities *in vivo* rapidly change, and taken in subsequent time intervals do not correlate with each other (Figure 6D).

### Modeling MT Growth

The analysis of the correlation between the comet length and microtubule growth rate is not straightforward. We obtain the comet tail length directly from one snapshot, while the velocity comes from a measurement of displacement between two consecutive snapshots. It means the velocity is measured between the moments when the tail length is determined, generating uncertainty when both values are stochastically changing. Thus, a more accurate result will be obtained when determining a correlation between averaged values of velocities and/or tail lengths. Addressing this question, we developed the computer model of microtubule growing. We use a model with parameters that can be found experimentally by developed code, which simplifies it compared to the one proposed earlier for *in vitro* measurements (Rickman et al., 2017).

In our model, the MT tip is considered as a 1D object which grows along the axis x. The velocity of growth is a random variable with normal (Gaussian) distribution. This statement has good agreement with experimental data (Figure 6B) and *in vitro* data (Vasquez et al., 1997; Dragestein et al., 2008; Rickman et al., 2017).

The MT comet image could be decomposed into several “regions of interest” (ROI on Figure 6A). Each ROI is generated by a single snapshot taken by the camera and represents elongation of the MT tip during a time interval between successive snapshots. In the proposed model, every region has its length depending on the growth rate and intensity depending on its age.

We set the time discreet in our model equal to one. At zero time point, the length of the MT comet is zero, and the regions are built subsequently one by one following time discrete. The age of the first region in a sequence is assigned to zero, its length is t*V_rand_, where t = 1 is time discrete and V_rand_ is velocity equal to some random value distributed normally. The coordinate of the MT end becomes X_1_ = X_0_+ 1*V_rand_. During the next period, another region is added, and the previous region will fade. This new region has zero age, but for the previous region, I will now have age 1. This process will continue, and in the time period, n coordinates of the MT end will be X_n+1_ = X_n_+ V_rand_. Because of the gradual disassembly of EB-3 from the tubulin (MT wall) behind the MT tip (Galjart, 2010), the intensity of each region will decrease with time until it drops below the threshold.

At some moment n, we will have X_n_ sequence of all regions that are above the threshold (Figure 6A). The age of the first region is always zero. The number of regions might be less than n because of negative velocity (MT shortening). For every region, the concentration of EB-3 decreases along with its age by exponential law:

$$n\left(\text{T}\right)=\;e^{-\frac{T}{\text{DT}}}.$$

The half-time of the existence of EB-3-binding sites at microtubule plus ends is termed “decoration time” or DT (Bieling et al., 2008). Time (T) in our model is determined as a discrete function as the age of ROI. So, the dependence of the concentration of EB on the x coordinate as continuous function could be described as follows (Figure 6B, orange line):

$$n\left(\text{X}\right)=\;e^{-\frac{T\left(X\right)}{\text{DT}}}.$$

Analysis of the comet length and displacement requires precise determination of the real position of the MT tip.

Assuming the growth velocity is constant, the intensity of a given region of the comet is equal to maximal intensity divided by DT (decoration time). However, the real value deviates from it because of stochastic variations. The question is how to determine the position and real intensity of the comet maximum. The comet intensity profiles were previously approximated by Gaussponent (Mustyatsa et al., 2019). Because of the image convolution by the microscope, the position of the exponent maximum in this model is localized within the “Gaussian” part. Our model takes into account that the MT tip is shifted from the brightest point on the intensity profile because of the image blurring by the microscope. The amount of shift depends on the comet brightness (Maurer et al., 2014) and is relatively large (319 ± 164 nm for HT1080 cells).

Another ambiguity comes from measurements of the comet’s velocity. The comet profile is obtained by summing the signals from all fluorescent molecules for the exposure time. When velocity is nearly constant for a long time, the intensity profile could be determined by using frame averaging (Rickman et al., 2017). When apparent velocity fluctuates significantly, averaging might give biased results. To overcome the possible problem caused by superaveraging, we developed the model, allowing determination of the full length of an individual comet and position of its maximum with subpixel precision. Analysis of MT growth velocity performed in a standard way and taking into account the real position of the MT tip (according to Figure 5E) show that velocity in HT1080 cells could be well-approximated by Gaussian distribution (Figure 6C). It is important to notice that velocities determined in subsequent short time intervals (0.5 s) do not correlate with each other (Figure 6C).

### On the Correlation Between Comet Tail Length and MT Growth Velocity

**FIGURE 7:**
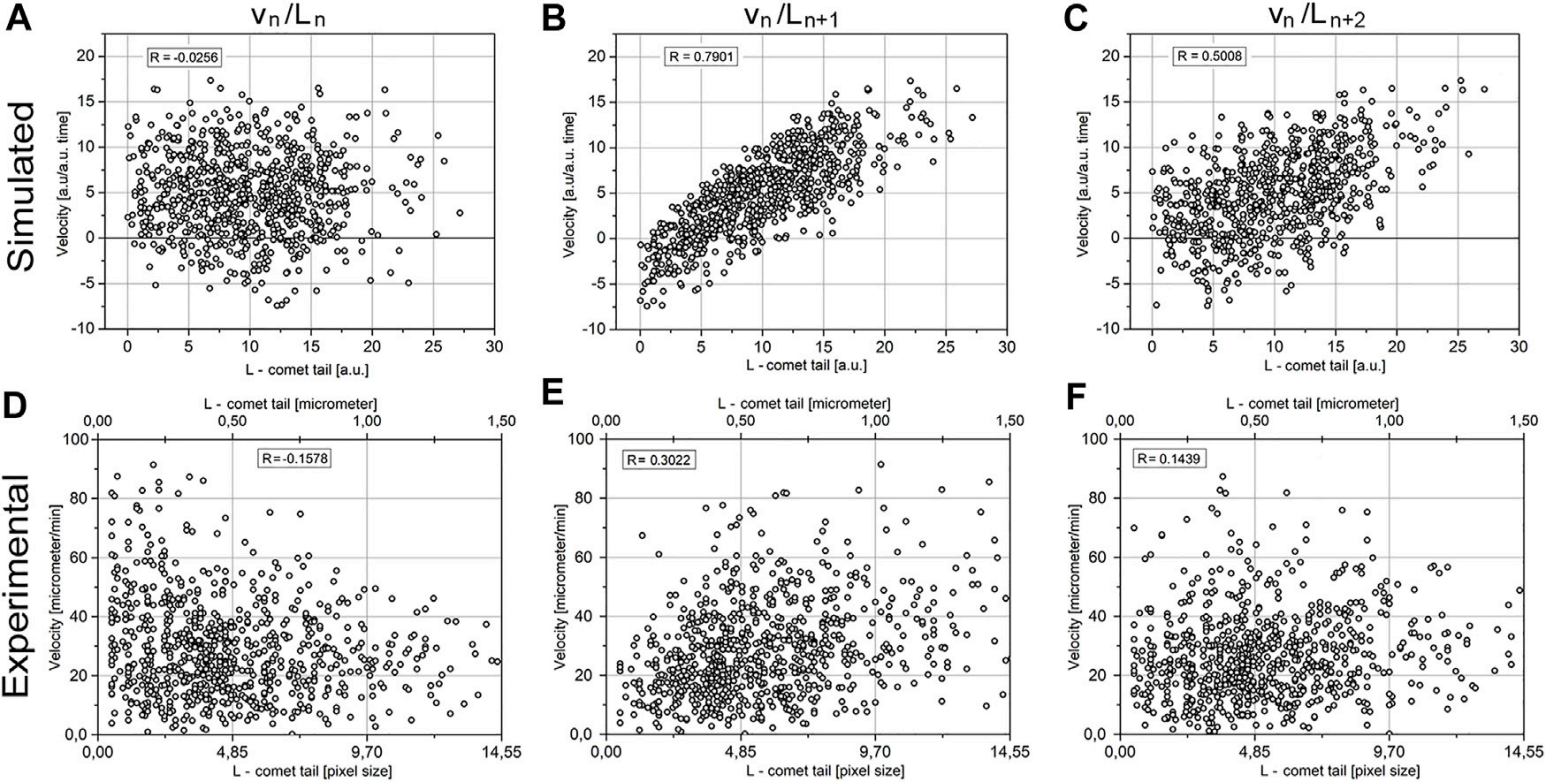
Correlation between the velocity of MT elongation and tail length. The comparison of the simulated model data **(A−C)** and the experimental data **(D**–**F)**. Images **(A, D)** represent the correlation between *v*_n_ and L_n_, image **(B, E)** represents *v*_n_/L_n+1_, and **(C, F)** represents *v*_n_/L_n+2_.

Another ambiguity comes from measurements of the comet’s velocity. The MT growth velocity is calculated from the distance between two consecutive measurements, while comet length is determined only at the beginning and the end of this period. It causes uncertainty in determining a correlation between the velocity (*v*_n_) and comet length (L_n_) (Figure 7). The average velocity of MT growth measured *in vitro* remains nearly constant over long periods—more than 30–40 s (Rickman et al., 2017), while *in vivo* usually only relatively short tracks of MT growth could be visualized and instantaneous growth velocity seemingly varies (Vorobjev et al., 1999; Matov et al., 2010).

Addressing this question, we analyzed the correlation between *v*_n_ and L_n+t_, where t is the time interval against *v*_n_. Taking into account a relatively large dataset (more than 6,000 events), we assumed that all values of the Pearson coefficient R > 0.1 will be significant. The model predicts no correlation between *v*_n_ and L_n_, the highest correlation between *v*_n_ and L_n+1_, and decreased correlations between *v*_n_ and L_n+k_, where k > 1 (Figures 7A–C).

The experimental data are in good agreement with the model. There is maximal R = 0.3 for *v*_n_/L_n+1_ for experiment (Figure 7E) and R = 0.79 for model (Figure 7B). For *v*_n_/L_n+2_ both for experiment and model there is reducing correlation: experiment R = 0.14 (Figure 7F) and model R = 0.5 (Figure 7C). There is good agreement between experiment and model.

The modeled data have higher coefficients of correlation than experimental ones since the model is ignoring errors in measurements. Also, in the model, MT is assumed to grow along the straight line, that is, in one dimension, while in the experiment, MT growth deviates from the straight line and occurs in the 2D space.

**FIGURE 8:**
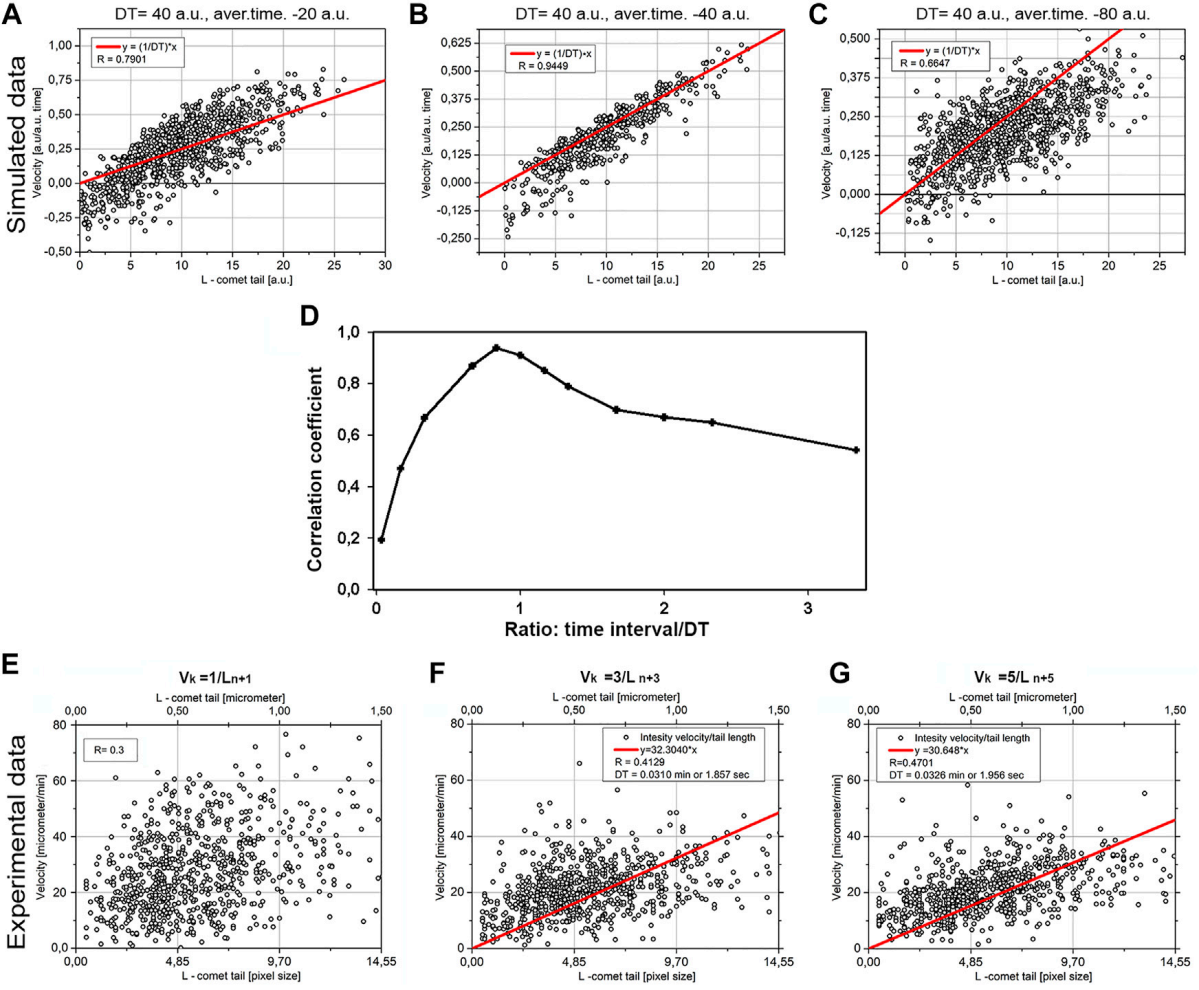
Correlation between tail length and averaged velocity of MT elongation. The comparison of the simulated model data **(A**–**C)** and the experimental data **(D**–**F)** retrospectively. The model **(A)** DT = 40 a.u., averaging time = 20 a.u., **(B)** DT = 40 a.u., averaging time = 40 a.u., **(C)** DT = 40 a.u., averaging time = 80 a.u. The correlation of tail length/velocity for v_k_ = 1/L_n+1_ in experimental data **(E)** the correlation (R) for v_k_ = 3/L_n+3_ **(F)**, and correlation for v_k_ = 5/L_n+5_ **(G)**. For **(F, G)** the calculated regression line y = ax also shown.

The only significant discrepancy between the model and experimental data is correlation *v*_n_/L_n_, where simulation of the model data gives R = −0.02 that is below the level of confidence (Figure 8A), while in experimental data, the correlation between *v*_n_/L_n_ in an experiment has a negative value of R = −0.15 (Figure 8D) that is by a module larger than the confidence level. This means an inverse correlation between the comet length and growth velocity of MTs in the following period of time exists in the cells.

### Finding Decoration Time for EB Protein on MTs *in vivo*

For the MTs, growing *in vitro* decoration time was found by dividing the comet tail length by the MT growth speed, that is, as a coefficient of the slope of line L/*v* (Bieling et al., 2008). However, for the *in vivo* data we have obtained, both parameters (L and *v*) are fluctuating, and the maximal correlation coefficient R is equal to 0.3 (Figure 8E), precluding finding the coefficient of the slope with reliable precision (i.e., with large S.D.-to-mean ratio). Thus, another approach was needed. Our model predicts that maximal length/velocity correlation occurs when the time interval for the velocity measurement is close to the DT value (Figure 8D), and comet length is taken from the frame after the interval where velocity was determined.

The averaged velocity of MT growth was obtained by skipping intermediate planes: we calculated velocities from the shifts of MT tip between planes (time points)—z and z+2, z and z+3, etc. (Figure 8). Denoting k as a distance between sequential planes, the average velocity will be as follows:

$$\vartheta_{k}=\;\frac{\sqrt{{\left(x_{n}-x_{n+k}\right)}^{2}+{\left(y_{n}-y_{n+k}\right)}^{2}}}{k\text{∗}t},$$

where $\sqrt{{\left(x_{n}-x_{n+k}\right)}^{2}+{\left(y_{n}-y_{n+k}\right)}^{2}}$ are distances obtained for different *v*_k_ (Figure 8D), t defines the time interval between successive frames (500 ms), and *v*_k_ is velocity averaged for the time interval k*t. Should note that higher k required a long trajectory for a comet. For k = 5, for example, the comet must have no collision with any other comet or any other object for 2.5 s. As a consequence, the number of these comets is not very large. For example, the number of points for k = 5 is about 800, for k = 6 about 300. In this case, we need to keep the balance between a good statistic and good correlation coefficients.

The result for modeling is shown in Figure 8. Three cases are shown: when time-averaged is less than DT (Figure 8A), when averaged time is equal to DT (Figure 8B), and averaged time exceeds DT (Figure 8C). The maximal correlation value (R = 0.9449), shown in Figure 8B, means that averaging time represents DT. The overall dependence of correlation coefficient versus time/DT ratio in the model is given in Figure 8D. Thus, the model confirms the applicability of the method for estimating DT as the coefficient of a slope when R reaches its maximum.

We next plotted experimental data *v*_k_ versus L_n+k_ for different k values (Figures 8E–G). The maximal R coefficient (R = 0.47) was obtained for k = 5, which means the averaging time is 2.5 s. Linear regression y = *a*×*x* was made fitting the data on Figures 8F,G. This regression gives the coefficient of the slope a = 0.51–0.53. DT in this case will be DT = 1/a so DT = 1.86–1.96 s.

The increased coefficient of correlation with k is in good agreement with the model. In this case, the model predicts the maximum correlation for k equal or slightly greater than DT (Figure 8D). The found DT is about 2 s, which corresponds to k = 4, that is, k = 4∼5 will be more or less optimal for our case. A further increase in k leads to a decrease in statistics—for k = 6, we will have only 300 points; for k = 7, only 170; and so on. Such small statistics will reduce the reliability of the data.

### MT Growth Under Nocodazole Treatment

**FIGURE 9:**
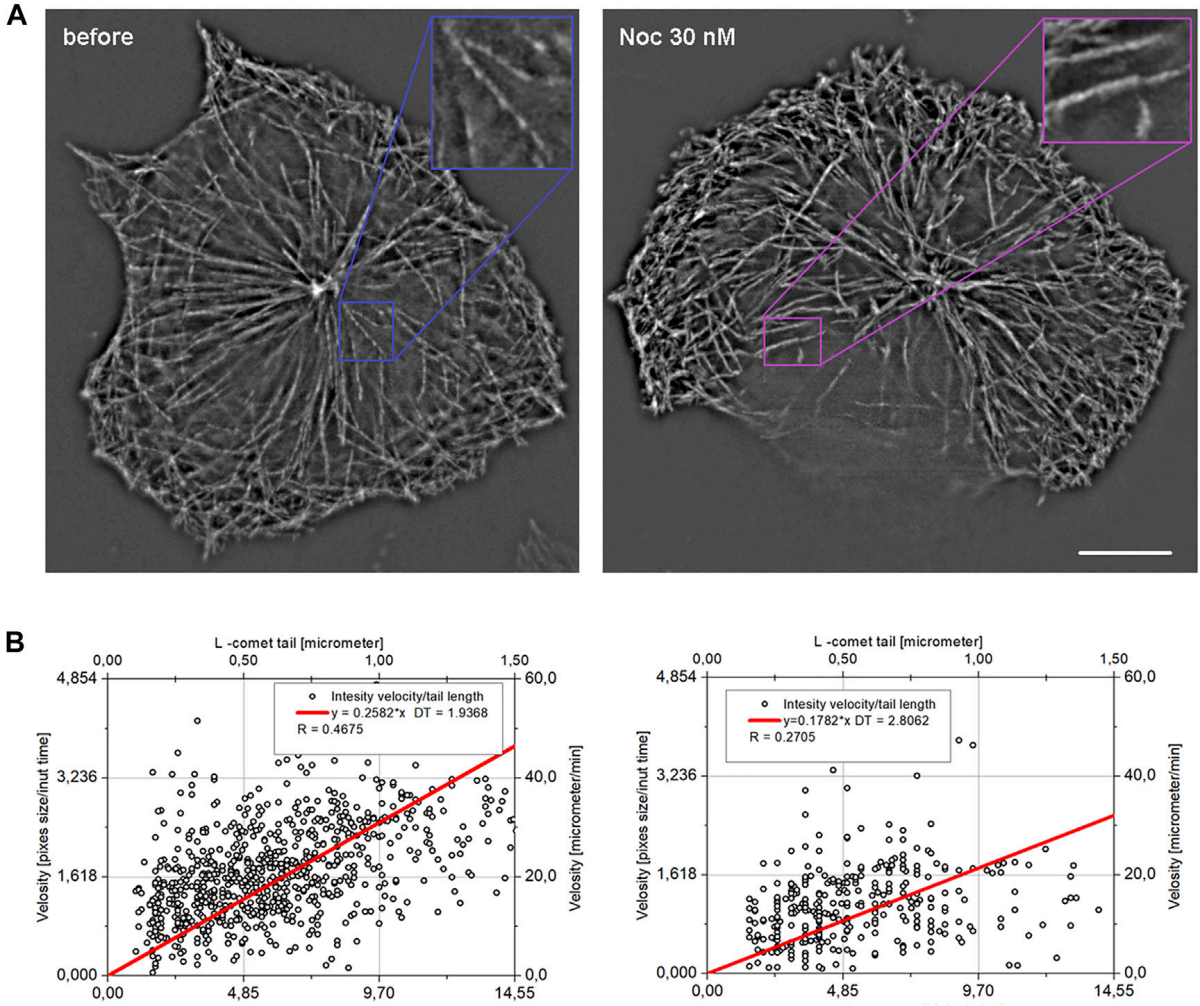
Projections showing MT growth in HT 1080 **(A)** cells before and after treatment with nocodazole. Images were taken for 2-s intervals (each 4th frame) and merged as maximal intensity projection. In the internal cytoplasm, faster translocation of the EB comets in the control cell is evident (enlargement). The length–velocity correlation **(B)** for k = 4 (*v*_k=4_ versus L_n+4_, 2 s interval) for control cells and cells after nocodazole treatment are shown.

We further analyzed the effect of nocodazole applied in nanomolar concentrations. In the range of concentrations, 30–50 nM, the velocity of MT growth estimated by manual tracking did not significantly change (Serikbaeva et al., 2018). However, in some cases, smaller displacements of individual MT ends were evident (Figure 9A). Automated analysis using a much larger sample demonstrated a decrease in the mean velocity −236 ± 0.150 nm/s compared to 329 ± 171 nm/s (19.74 µm/min) in control cells (*p* < 0.0001) and an even more profound decrease in the comet length –525 ± 245 nm compared to 1,252 ± 624 nm in control (*p* < 0.0001).

Since the maximum correlation between comet tail length and growth velocity will be when velocity is averaged on time ∼ DT, we used averaged velocity on k = 4 that is relate to 2 s (Figure 9B,C). The R coefficient = 0.27 obtained after treatment is higher than the confidence level (for 300 cases it is about 0.2), but rather low compared to untreated cells. Based on these data, DT is 2.8 s (determined from the linear approximation slope). Thus, we conclude that DT for EB-3 is the same for reference and nocodazole-treated cells. EB comets were also found at higher nocodazole concentration, but MTs were nearly stationary under such treatment, and it was not possible to determine the L value (data not shown).

## Discussion

The developed model for MT comet approximation from individual images of plus end comets and new MT tip tracker shows good capabilities to extract the spatial and temporal characteristics of MT comets in living cells. The advantage of our model is that it gives the real position of the MT tip for a given comet with the accuracy not achieved before for the *in vivo* imaging. Our algorithm of comet length determination is similar to the Gaussponent approximation developed previously (Mustyatsa et al., 2019) but takes into account the shift of the real comet tip from its apparent position in the microscopic image (Maurer et al., 2014, and Figure 4F).

Theoretically, the position of the MT tip could be calculated precisely from the deconvolution of the comet image using the microscope point spread function (PSF) (Maurer et al., 2014). However, iterative deconvolution requires 3D imaging (Dias-Zamboni et al., 2007) or could be applied only to relatively homogenous datasets (Maurer et al., 2014; Rickman et al., 2017). Data for successful deconvolution of MT comet profiles were obtained by superaveraging of hundreds to thousands of individual images recorded *in vitro* (Maurer et al., 2014; Rickman et al., 2017). This approach cannot be used *in vivo* because the MT elongation velocity at the population level in the cultured cells is not constant, instead varies significantly even within one cell (Serikbaeva et al., 2018).

Our observations show that approximation of the comet profile with subpixel resolution could be achieved without averaging. Thus, it was possible to determine the velocity of MT growth with higher precision than the previous analysis when comet displacement was determined as the distance between maximal intensity pixels in the comet profile in sequential images (Matov et al., 2010; Applegate et al., 2011; Serikbaeva et al., 2018).

Measurements of MT growth velocity in the current study show that despite the heterogeneity of the actual velocity of MTs, correlation between velocity and length of an EB comet determined for individual growing MTs *in vivo* is significant. The maximum observed coefficient of correlation is equal to R = 0.47 when the time of averaging was about or slightly longer than decoration time (DT). In the HT1080 and U-118 cells, the decoration time is about 2 s, and the best correlation between *v*_k_ and L_n+k_ is observed for k = 4∼5 (k is a time discrete, which in our case is 0.5 s). This coefficient is highly significant. Good agreement between model and experimental data, in general, confirms the mechanism of MT growth that was earlier proposed for *in vitro* conditions (Galjart, 2010).

This suggestion is confirmed by observations of cells treated with a low dose of nocodazole (30 or 50 nM). Under nocodazole treatment, significant reduction of both growth velocity and comet length occurs, velocity drops down to 236 ± 150 nm/s compared to 329 ± 171 nm/s (19.74 um/min) in untreated cells (*p* < 0.0001), and the comet length shrinks to 525 ± 245 nm against 1,252±624 nm in untreated cells (*p* < 0.0001). The correlation of velocity and comet length after treatment with nocodazole exists, and DT seems to be the same as without treatment.

MT instantaneous growth velocity distribution determined *in vivo* fits in the normal distribution (Figure 6B), and velocities determined in subsequent time intervals do not correlate with each other (Figure 6C). The decoration time for EB-3 assembly on the growing MT tip for HT1080 cells is 2–2.5 s, which is significantly shorter than *in vitro* (Bieling et al., 2008). We suggest that faster accumulation of EB proteins on the growing MT tips *in vivo* than the *in vitro* measurements is achieved due to the action of additional plus TIPs, such as CLASPs facilitating assembly of EB proteins with polymerized tubulin (Watanabe et al., 2009; Grimaldi et al., 2014).

## Conclusion

Automated unbiased tracking of MT plus ends decorated with EB-3 shows that elongation of individual MTs in cultured cells could be approximated by the constant velocity with random fluctuations and thus do not differ from the dynamics of MTs observed *in vitro*. Large variations of the instantaneous growth velocity *in vivo* compared to the *in vitro* measurements could be explained by the shorter decoration time. Shorter decoration time *in vivo* could be explained by the presence of other decorating proteins like chTOG, assembling at MT tip in living cells before EB (Nakamura et al., 2012). Growth velocity measured at time intervals close to the decoration time and comet length taken at the end of this period correlate with each other, and this correlation remains under low-dose nocodazole treatment when both velocity and comet length is significantly reduced.

## Materials and Methods

### Cell Culture, Transfection, and the Microtubule Drugs

Experiments were performed on HT1080 and U-118 cultured cells. Cells were maintained at DMEM supplemented by 10% fetal bovine serum in the presence of 4–8 µm of L-glutamine and 100 U/ml penicillin/streptomycin mix. The transfection was performed with Evrogen plasmid (Cat. No. FP142) carrying EB-3-TagRFP fusion protein (EX.555 nm/EM.584 nm) with Xtreme ROCHE transfection reagent (Sigma, Cat. No. 6365787001) according to the manufacturer’s protocol. The cells were maintained in the standard growth media. Nocodazole (Sigma, Cat. No. M1404-10MG) in the concentrations of 30 and 50 nM was used as a microtubule growth inhibition drug. For imaging, cells were subcultured onto 8-well glass-bottom plates (Nunc Lab-Tek II, Thermo Cat. No. 155409), and transfection was performed on the following day. The culture medium was replaced 24 h post-transfection, and microscopy recording was performed 36–48 h after transfection.

### Live Cell Imaging

Fluorescent microscopy was performed on a Cell Observer SD microscope (Carl Zeiss GmbH), equipped with a heating incubator chamber. Imaging was performed on cells with an appropriate level of transfection without overexpression patterns. Comets chosen were resolvable from the background, and measured signal intensity was at least 4–5 times higher than the background of the cytoplasm in an area of 10–50 pixels of an image. These cells were recorded as a control in imaging CO_2_-independent DMEM growth medium, and then coordinates of each cell were stored for further steps. After the media was carefully replaced with a drug-containing culture medium and cells were exposed for 60 min on the microscope stage, the temperature of 37°C was maintained by heating the incubator chamber. Imaging of treated cells was performed according to obtained coordinates to have valid pair of control (cell before treatment) and experiment (same cell after addition of nocodazole).

To obtain maximal intensity without photobleaching of comet during time-lapse, image acquisition was performed at a single focal plane in the wide-field fluorescence mode or using spinning disk confocal microscopy. Image sequences were recorded with 500-ms step, with an exposure time of 300 ms using an HXP120 metal halide lamp and Rhodamine filter cube (Filter set 43 HE) and Hamamatsu ORCA V2 sCMOS camera (2,048 × 2,048). Some image samples were collected using a Yokogawa CSU-X1 spinning disk confocal unit using a 561-nm laser and Rolera EM-CCD detector (1,024 × 1,002). Imaging in both setups was performed with Plan Apochromat 63x/1.46 oil immersion objective, and the image pixel size was 103 nm. On five separate experimental days, we recorded time-lapse videos of HT-1080 and U-118 transfected cells. Images were obtained using Zen software and exported as 16-bit TIFF uncompressed image sequences for processing. The original tiff image stacks were cropped manually to the margins of the individual cell which contained clearly visible microtubule comets. Each image stack contained 100 images. The number of individual cells (recorded before treatment and recorded the second time after nocodazole treatment for >30 min) for each experimental day was about 50–100. The image sets obtained after treatment with nocodazole at concentrations of 30 and 50 nM were further merged, while images obtained under nocodazole 100 nM treatment were excluded from analysis due to significant shrinkage of EB comets precluding measurement of their length. In total, we collected 486 image sequences of individual HT1080 cells before treatment and 328 cells after nocodazole treatment (30 nM + 50 nM). Further analysis was performed in MATLAB and C++ environments, as described in the Results section. Cells, where photobleaching was significant, were discarded from automated analysis. The tool and commentary uploaded in https://github.com/astrv-103/tracker.

## Data Availability

The raw data supporting the conclusions of this article will be made available by the authors, without undue reservation.

## Supplementary Material

Supplementary Video S1 Related to Figure 1. Time-lapse sequence of fluorescence images of EB-3 comets translocation.
