# Supplementary material for: On the Relationship Between EB-3 Profiles and Microtubules Growth in Cultured Cells
Source: Front Mol Biosci. 2021 Nov 8;8:745089. doi: 10.3389/fmolb.2021.745089 (PMC8606533; doi:10.3389/fmolb.2021.745089)
Supplement: Supplementary file 2 [file DataSheet1.docx]

**On the relationship between EB3 profiles and microtubules growth in cultured cells**

Arshat Urazbaev1, Anara Serikbaeva1,*, A. Tvorogova2, A. Dusenbayev1, S. Kauanova 3, Ivan Vorobjev 1,2,3**.

**Supplementary material.**

Gradient method is now the standard method to determine the boundaries of the object (comet in our case). Usually the boundaries of the object are pixels whose amplitudes differ from the amplitude of neighboring pixels by certain (selected) value. In general, the method is equivalent to a simple flood fill algorithm (Fig.2). Any raster pattern consists of a pixel 2D matrix, where each pixel has its own intensity value.

We will use term “Point cloud”. This is a set of points in 3D space (x,y coordinates, and intensity). Because image is matrix, x and y coordinates are row and column number). If most of these points are neighbors to each other they form single object.

0

10

0

12

0

14

0

10

0

11

0

6

11

72

0

0

0

10

5

5

0

10

60

70

6

10

50

66

58

79

60

59

60

54

72

80

62

51

60

80

90

50

Pixel belonging to the

point cloud object

All neighbor considered pixels

Pixel of matrix of intensities

Considered pixel

Suppl. Figure 1. Scheme of 2D flood fill algorithm.

All background pixels have intensities equal to zero after preprocessing. Only pixels with non-zero intensity will be further considered in our operations. Since background pixels are majority of all pixels in the image (Suppl. Fig. 1 B) it makes computation faster.

The Algorithm is:

At the initial moment, a pixel with certain amplitude is considered. Next step: we consider all eight neighboring pixels:

1) If this pixel already in point cloud object then return,

2) If pixel have zero intensity (background) then return,

3) If the difference in the amplitude of pixel exceeds a certain magnitude of Г comparing to original pixel then return

2) If the difference in the amplitude of pixel does not exceed a certain magnitude of Г compared to the original pixel, then this pixel is attached to the point cloud object. Then this process is repeated recursively for this pixel.

Г value is characteristic gradient or difference between intensity of pixels on the border of the object. To find Г value let’s consider the point object. In microscopic image it will appears as an Airy disk. Intensity distribution in the Airy disk can be approximated by Gaussian one [5]. Taking into account the size of Airy disk obtained by oil immersion objective with NA=1.4 and wavelength of emitted light 570 nm the diameter of Airy disk will be 0.61*580/1.4=252.7 nm and equivalent pixel value of the camera of 106 nm, half width of the Gaussian fit will be d = 3 pixels.

The average intensity of comets in our images can be found by

$G= mean(\max\left( M \right))$,

Where M is a 2D matrix of intensity of our microscopic image. Function max(M) will be function that finds all local maxima in the matrix. G in this case will be averaged amplitude of all comets. Let us further consider Gaussian with half width equal to 3 pixel, and amplitude G. The maximum gradient (difference between two neighbor pixel) will be 0.14*G. In our case G is equal to 173 A.U. So 0.14*G will be equal to 24 A.U. To find border of this point object Г must be slightly lower than maximum gradient. Seventy percent of this value was used as Г. So Г = 0.7*0.14*173 = 15 was used.

Example run of algorithm shown in figure 2: Matrix pixel is violet squares, and point cloud pixels are marked green. Blue square is considered pixel and it has intensity 70. It has eight neighbor pixels (red squares). Downer pixel is already belongs to point cloud, so there will be return. Left pixel has intensity 6, so 70 - 6 = 64 > Г (Г = 15 in our case). So there also will be return. For upper –right, upper, upper-right pixels will be the same.

Righter pixel has intensity 60. Difference will be 70 – 60 =10 < Г. This pixel will be attached to pixel cloud, and at next step this pixel will be considered pixel, and also eight neighbor pixel will be also considered for this pixel. For downer-right, and downer-left pixels will be the same procedure.

Result of this operation will be point cloud object where every pixel has intensity difference with its neighbor’s pixels less than Г. If we replace all pixels in matrix belonged to the cloud by one, and all non-cloud pixels by zero the binary mask of the object will be obtained. It will be very close to the binarization operation. But this method can give better results comparing to the threshold binarization: for example you have picture of apple and part of this apple in shadow. Threshold will cut this part of apple, while gradient method can give you the right result.

This method allows quickly determining the border of the object, and subsequently the elongation of an object. This elongation depends on comet tail value. Unfortunately, it doesn’t work well due to the fact that boundaries were pixels with low intensity. These pixels are highly affected by the noise. And determination of the borders of elongated object is not precise enough.


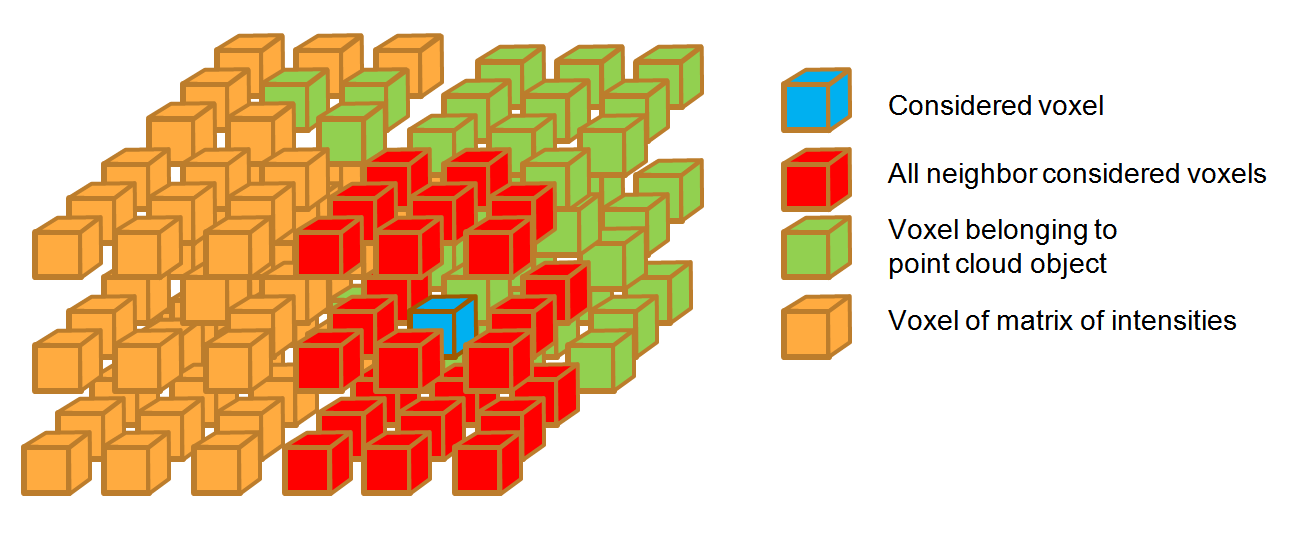


Suppl. Figure 2. Scheme of 3D flood fill algorithm.
